# Preparation and properties of cellulose nanocrystal-based ion-conductive hydrogels

**DOI:** 10.1039/d2ra04660a

**Published:** 2022-12-22

**Authors:** Xinmin Huang, Xiang Ao, Lianhe Yang, Jing Ye, Chengwei Wang

**Affiliations:** College of Textile & Clothing, Yancheng Institute of Technology Jiangsu 224051 P. R China huangxinmin@126.com; School of Textile & Science Engineering, Tiangong University Tianjin 300387 P. R China

## Abstract

Ion-conductive hydrogels were prepared by a simple one-pot method based on cellulose nanocrystals (CNC) and polyvinyl alcohol (PVA). PVA–CNC hydrogels were prepared with different contents of CNC and Al^3+^ ions to enhance the performance of ion-conductive hydrogels. The samples were characterized by Fourier transform infrared spectroscopy, universal testing machine, LCR digital bridge and scanning electron microscopy analyses. The results show that DMSO solvent can enhance the anti-freezing and moisture retention property of the polyvinyl alcohol hydrogel. With the increase of CNC content in the hydrogels, their mechanical properties are also improved. When the CNC concentration is 0.2 wt%, the maximum tensile strength and elongation at break are 750 KPa and 410.47%, respectively. Compared to the hydrogel without CNC, the tensile strength of the hydrogel with 0.2 wt% CNC was increased to 733% and elongation at break was increased to 236%. However, the mechanical properties of the hydrogel will decrease when the CNC content increases to 0.25 wt%. When the hydrogel is stretched, the relative resistance of the hydrogel increases with the increase of tensile deformation. The hydrogels can also be assembled to form self-powered batteries with a voltage of 0.808 V. This indicates that the hydrogels have potential application value in flexible sensors.

## Abstract 2

Ion-conductive hydrogels were prepared by a simple one-pot method based on cellulose nanocrystals (CNC) and polyvinyl alcohol (PVA). PVA–CNC hydrogels were prepared with different contents of CNC and Al^3+^ ions to enhance the performance of ion-conductive hydrogels.

## Graphical abstract

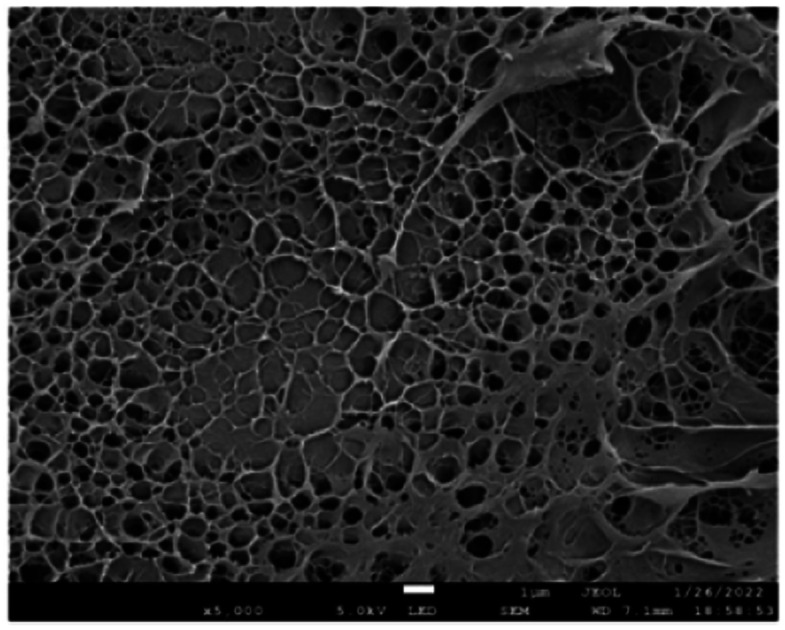

## Introduction

Hydrogels are soft polymer materials composed of hydrophilic polymers and have a three-dimensional network structure, with excellent flexibility, water retention, and good mechanical properties. A conductive hydrogel is an organic combination of a hydrophilic matrix and conductive medium, forming a new type of composite hydrogel with high flexibility, good electrochemical performance and processability.^1^ Generally speaking, the traditional hydrogels have been unable to meet the needs of practical applications. Therefore, many researchers began to incorporate different materials into hydrogels, such as conductive polymers,^2^ polymer reinforced materials,^3,4^ adhesive materials,^5,6^ bioactive substances and antifreeze materials,^7,8^ making hydrogels have different functions.

Nowadays, there are more and more studies on nanocellulose. Cellulose nanocrystals have many advantages, such as large surface area, high mechanical strength, low thermal expansion rate,^9^ biodegradability, biorenewability, low toxicity and so on. PVA is colorless, flammable, odorless and tasteless, transparent and white granular fine particles. The polymer's capabilities make it easy to use in a variety of applications, such as the paper, wood, leather, textile industries, cosmetics and agribusiness.^10,11^ In terms of biological applications, PVA can be prepared into hydrogel by the freeze-thaw method.^12^ The hydrogel has good flexibility, but poor rigidity, which cannot meet the requirements of high strength application. The mechanical properties of PVA hydrogel can be enhanced by adding fillers and organic solvents.^13,14^

In this paper, a one-pot method was used to prepare PVA/CNC organic hydrogel. The PVA and AlCl_3_ were dissolved in the mixture of CNC suspension and DMSO solvent with different concentrations. The magnetic agitators were used to dissolve all of them, and the hydrogel was prepared after repeated freezing and thawing. The performance of hydrogels with varying concentrations of CNC and the conductive performance of hydrogels prepared with different ion concentrations were compared. The mechanical properties, electrical conductivity and sensing properties of PVA/CNC hydrogels were evaluated. The effects of the addition of organic solvents on the antifreeze and water retention properties of hydrogels were analyzed.

## Materials and methods

### Materials

Polyvinyl alcohol (PVA-1750 ± 5) was acquired from Sinopharm Chemical Reagent Co. Dimethyl sulfoxide (DMSO), AlCl_3_·6H_2_O and CaCl_2_ were acquired from Shanghai Titan Technology Co., Ltd. Cellulose nanocrystals (CNC) were acquired from Qihong Technology Co., Ltd. The above experimental raw materials are chemically pure and have not undergone any pretreatment.

### Experimental methods

First, 10 g of a certain concentration of nanocellulose crystallite (CNC) suspension was prepared, then 10 g of DMSO solvent was added to the CNC suspension, and the mixed solution was stirred on a magnetic stirrer for 10 min to make it fully mixed. 1.8 g of polyvinyl alcohol (PVA) particles and 0.6 g of AlCl_3_·6H_2_O particles were added to the above mixture and continued to be stirred uniformly with a magnetic stirrer. Then the mixed solution was put in a constant temperature magnetic stirrer, set the temperature to 120 °C, and stirred until the PVA particles are completely dissolved. After the mixed solution is completely dissolved, let stand for 10–20 minutes to remove air bubbles. The mixed solution were transferred to Petri dishes, frozen at −20 °C for 12 hours, removed, and thawed at room temperature for 12 hours. The thickness of the hydrogels were adjusted by controlling the amount of the mixed solution. The nanocellulose ionic conductive hydrogels were prepared by freezing and thawing three times. The PVA/CNC-0.1-Ca^2+^ hydrogel was prepared by replacing AlCl_3_·6H_2_O with CaCl_2_, the other experimental steps were the same. The composition of the hydrogels is shown in the Table 1.

**Table 1:** Composition of hydrogels

Sample	CNC (wt%)	PVA (g)	AlCl_3_·6H_2_O (g)	DMSO (g)
PVA/CNC-0	0	1.8	0.6	10
PVA/CNC-0.1	0.10	1.8	0.6	10
PVA/CNC-0.15	0.15	1.8	0.6	10
PVA/CNC-0.2	0.20	1.8	0.6	10
PVA/CNC-0.25	0.25	1.8	0.6	10
PVA/CNC-0.05-Al^3+^	0.20	1.8	0.24	10
PVA/CNC-0.075-Al^3+^	0.20	1.8	0.36	10
PVA/CNC-0.1-Al^3+^	0.20	1.8	0.48	10
PVA/CNC-0.1-Ca^2+^	0.20	1.8	0.28	10
PVA/CNC	0.20	1.8	0.6	0(10 g H_2_O)

### Scanning electron microscopy (SEM)

CNC hydrogels with different concentrations were freeze-dried to obtain dehydrated hydrogels. The dried hydrogel samples were sprayed with gold before the test, and the pore structure of the hydrogel was conducted by scanning electron microscope (FESEM, JEOL JSM-7600F, 5 kV).

### Fourier transform infrared spectroscopy (FTIR)

The samples were scanned by Fourier transform infrared spectroscopy (FT-IR), and the group changes of the samples were analyzed according to the absorption area of the sample groups. The resolution is ±2 cm^−1^, the wavenumber range is 400 to 4000 cm^−1^, and the step size is 4 cm^−1^.

### Mechanical test

The mechanical properties of the hydrogel sample (40 × 5 × 2 mm) were tested by an ST universal material testing machine at a loading speed of 20 mm min^−1^. The displacement–force and stress–strain curves were obtained.

### Electrical measurements

The ionic conductive hydrogel samples (40 × 10 × 2 mm) were quantitatively stretched and tested, and the relative resistance changes and sensitivity factors of hydrogels under different tensile strains were measured by a LCR digital bridge (VICTOR 4090C). Calculate the relative change of resistance (*R*/*R*_0_, where Δ*R* = *R* − *R*_0_), the sensitivity factor calculation formula is as follows (1):1
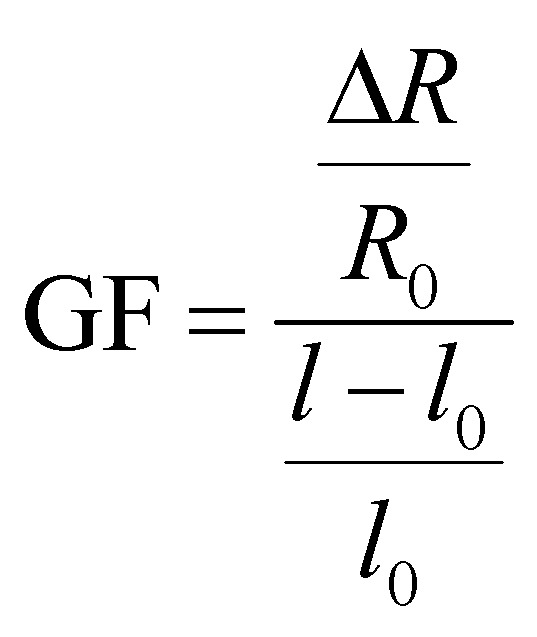


In formula: *l*_0_ and *l* represent the length (m) before and after strain application of the hydrogel; *R*_0_, *R* shows the resistance before and after strain (Ω) of strain.

### Moisture retention property test

The weight loss of the hydrogel was measured, and the relative weight of hydrogel (*W*_t_/*W*_0_ × 100%) -time curve was drawn (*W*_t_ is the mass of the hydrogel after being placed *T* (h), *W*_0_ was the initial mass of hydrogel).

### Anti-freezing property test

The hydrogel samples with and without DMSO were cut into strips and frozen at −20 °C for 24 h. The frozen hydrogel samples were used as electric conductors and connected to the circuit with an LED lamp to test the antifreeze performance of hydrogels.

### Thermal stability test

The thermal stability PVA/CNC hydrogel samples with CNC concentrations of 0 wt% and 0.2 wt% were assessed with thermogravimetric analysis. The sample was heated from room temperature to 700 °C at a rate of 10 °C min^−1^ in a nitrogen atmosphere.

## Results and discussion

### The structure of the hydrogel

**Fig. 1:**
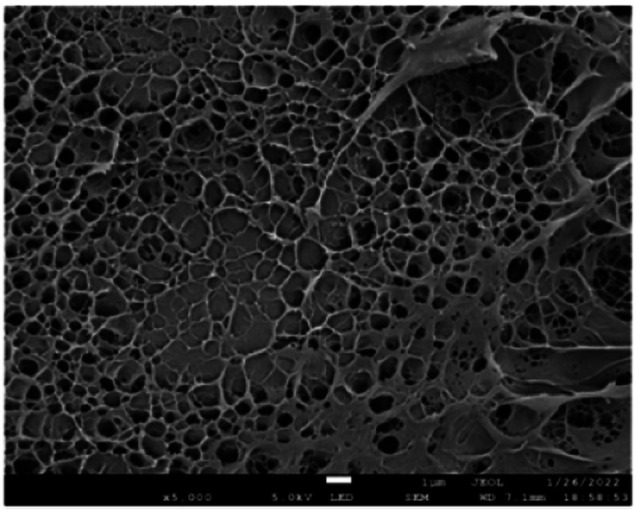
SEM image of the PVA–CNC hydrogel.

Fig. 1 is the SEM image of the PVA/CNC hydrogel, which reflects the cross-sectional appearance of the hydrogel. It can be seen from the image that the hydrogel has a three-dimensional network structure. The hydrogel presents a three-dimensional high-pore network structure formed by interconnected nanofibers. The size of the pores ranges from several nanometers to tens of nanometers, and the overall three-dimensional network structure of hydrogel shown in Fig. 1 is relatively uniform. Generally speaking, the nanofibers have a larger surface area, which makes the metal ions more likely to enter the cellulose in the network, which provides the conditions to improve the conductivity of hydrogel.

### FTIR analysis

**Fig. 2:**
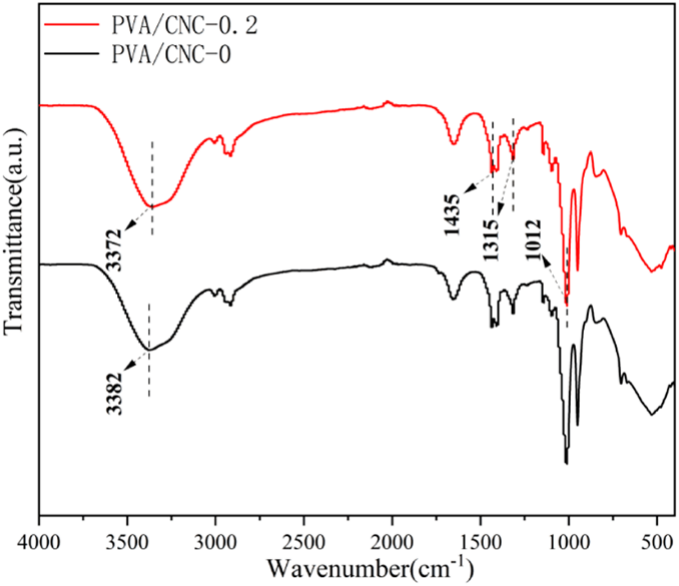
FTIR images of PVA/CNC hydrogels with different CNC contents.

As shown in Fig. 2, a broad and strong peak at 3372 cm^−1^ was related to the hydrogen bonding interaction between PVA and CNC. There are molecular interactions between nanocellulose and hydrogel, which mainly include van der Waals forces, hydrogen bonds and coordination bonds. The hydrogen bond vibration peak shifted from 3282 to 3272 cm^−1^ when the CNC content increased from 0 to 0.2%. It can be indicated that more hydrogen bonds was occurred in the PVA/CNC hydrogels due to the hydroxyl group on CNC. The exhibits absorption peaks due to the enhanced C–H bending vibration of the DMSO were at 1435 and 1315 cm^−1^ (ref. 15), while the stretching absorption peak appearing at 1012 cm^−1^ is assigned to the characteristic peak of S=O of DMSO. Therefore, it can be seen that DMSO and water molecules form a strong hydrogen bonding crosslink.

### Mechanical properties of hydrogel

**Fig. 3:**
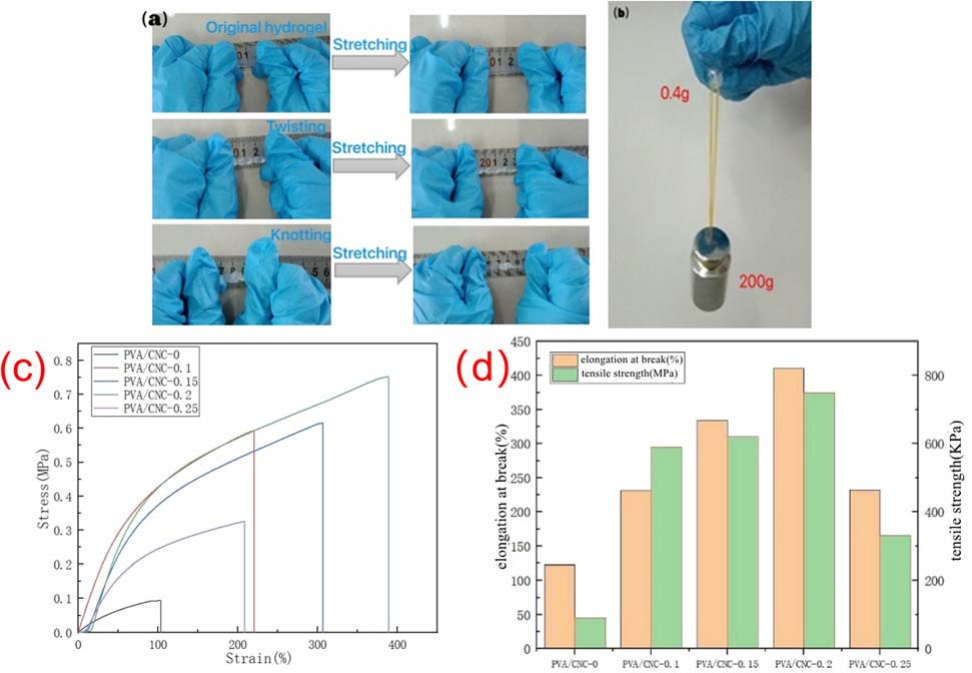
(a) Photos of hydrogel in original length, stretch, twist, twist stretch, knot and knot stretch (b) photos showing the PVA–CNC hydrogel can be stretched with lift weight (c) tensile stress–strain curves of the hydrogels with different CNC contents (d) the elongation at break and tensile strength of the hydrogels with different CNC contents.

As shown in Fig. 3, it can be seen that PVA/CNC hydrogel has excellent flexibility and mechanical properties. It can bear 200 g weight without breaking, that is, the hydrogel can bear weight 500 times larger than its weight (Fig. 3b). When the hydrogel is twisted and knotted, the hydrogel can remain intact, indicating the excellent mechanical properties of PVA/CNC hydrogel. The reason for the enhancement of mechanical properties may be due to a large number of hydroxyl groups on the molecular chain of CNC and PVA. The formation of hydrogen bonds between CNC and PVA can dissipate energy during deformation. The mechanical properties of the hydrogels were tested by a universal material testing machine, and the tensile stress–strain curves of the hydrogels with different CNC contents are shown in Fig. 3c. The elongation at break and tensile strength of the hydrogels are shown in Fig. 3d. The results show that CNC can improve the mechanical properties of hydrogels. With the increase of CNC contents in the hydrogel, the mechanical properties of the hydrogels were also improved. The hydrogel without CNC showed the lowest tensile strength and elongation at break, which were 90 kPa and 122.86%, respectively. Both the tensile strength and elongation at break of the hydrogels were improved after the addition of CNC. When the CNC concentration was 0.2 wt%, the tensile strength and elongation at break of the hydrogel reached the maximum value of 750 kPa and 410.47%, respectively. This is because the cross-linking of the PVA polymer chains in the nanocellulose and the hydrogel can form hydrogen bonds, and the CNC itself has a reinforcing effect in the polymer matrix, so the mechanical properties of the hydrogel can be significantly improved. However, when the CNC content increased to 0.25%, tensile strength and elongation at break of PVA/CNC-0.25 hydrogel started to decrease. This is because the high concentration of CNC will agglomerate or form more microcrystals in the hydrogel, resulting in the uneven distribution of CNC in the three-dimensional network structure, and the mechanical properties of the hydrogel will decrease. At the same time, due to the presence of Al^3+^ in the hydrogel, the coordination between CNC and Al^3+^ can also synergistically enhance the mechanical properties of the ion-conducting hydrogel.

### Electrical properties of hydrogel

**Fig. 4:**
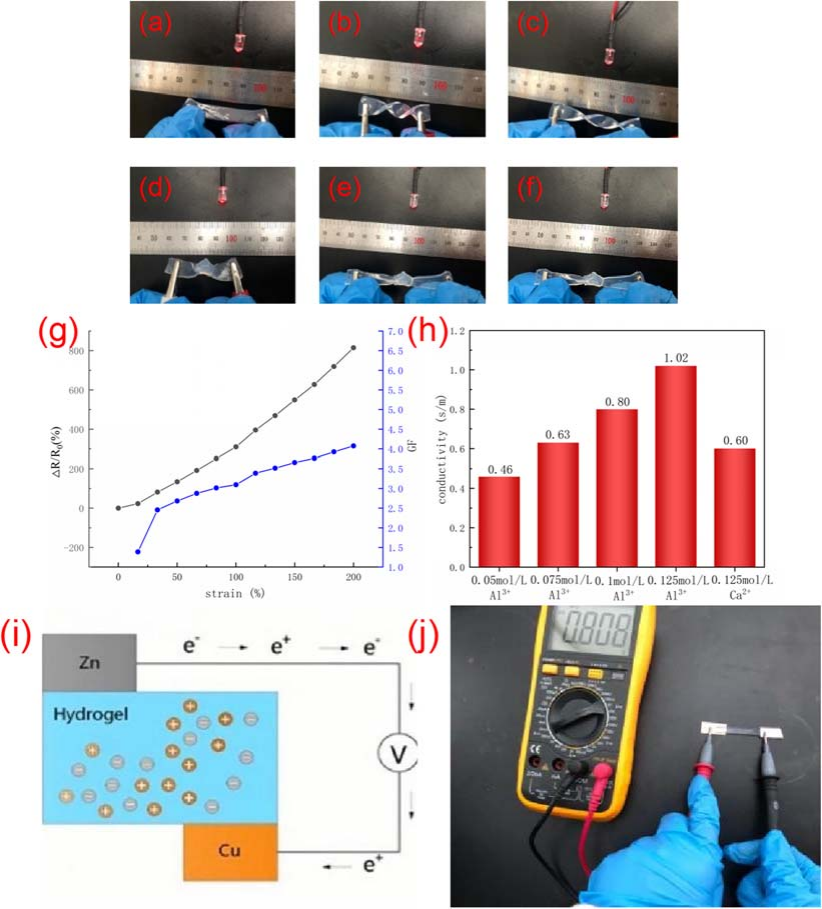
(a–f) Hydrogel connection in the circuit and through a variety of deformation and the change of the LED lamp brightness (g) resistance and sensitivity changes of hydrogels under tensile strain (h) conductivity of hydrogels with different ionic concentrations and metal ions (i) hydrogel was used as an electrolyte to form a self-powered device (j) photograph of the self-powered device.

When the ionic conductive hydrogel is used as a conductor and connected to the circuit, it can be found that the LED lamp can emit light normally. At the same time, when the ionic conductive hydrogel is stretched Fig. 4b, twisted Fig. 4c, twisted and stretched Fig. 4d, knotted Fig. 4e and knotted and stretched Fig. 4f, the LED lamp will also shine, but the brightness will change. As shown in figure Fig. 4g, the relative resistance change gradually increased with increasing strain. As shown in Fig. 4h, the hydrogels with different aluminum ion concentrations are connected to the circuit. It can be seen that with the increase of aluminum ion concentration, the electrical conductivity of the hydrogel will also increase. The experimental results show that the electrochemical performance of the hydrogel is affected by the concentration of ions, and the conductivity of the hydrogel is better with the increase of the concentration of aluminum ions. Fig. 4h shows that the conductivity of PVA/CNC-0.1-Ca^2+^ hydrogel is lower than that of PVA/CNC-0.1-Al^3+^ hydrogel. Due to the radii of these two ions (Al^3+^ < Ca^2+^), it is better for Al^3+^ ions to penetrate the holes in the three-dimensional network structure of hydrogel.

As shown in Fig. 4j, copper foil was used as the anode and zinc foil was used as the cathode. The hydrogel, zinc foil and copper foil can be assembled into a self-powered device, in which electrons flow continuously through conductors from the cathode to act as the anode, creating a potential difference. The hydrogel self-powered device can convert chemical energy into electrical energy. The experimental results show that the generated voltage is about 0.808 V(Fig. 4j). It can be seen that the self-powered sensor made of hydrogel can be self-powered without an external power supply, indicating that the hydrogel has a practical application prospect in wearable sensors.

### Moisture retention property

**Fig. 5:**
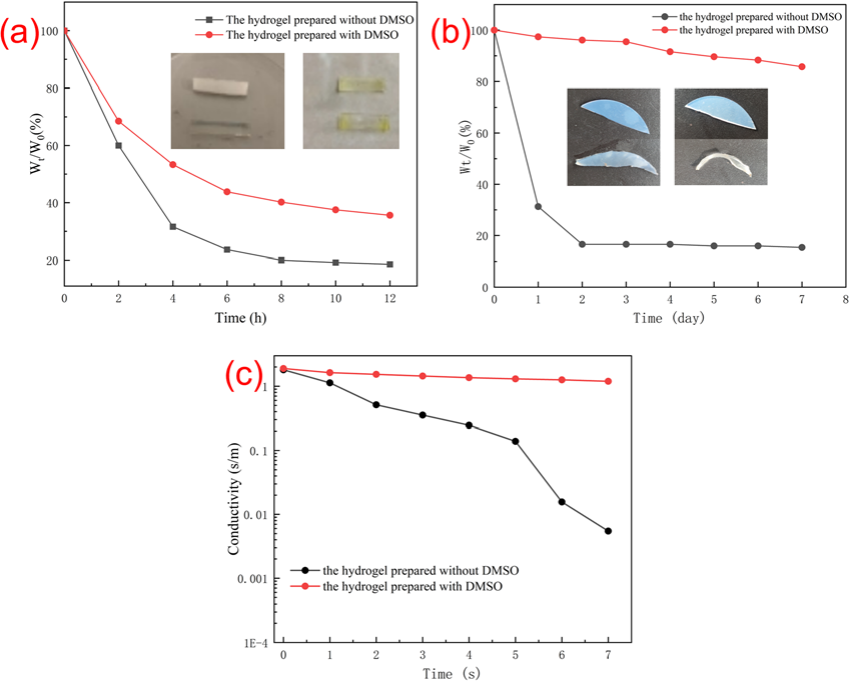
(a) The weight loss of the hydrogels in the oven was measured by every two hours. (b) The weight loss of the hydrogels in an open environment was measured each day. (c) Conductivity of the hydrogels after storage for 7 days.

The water-retention performance of the hydrogels was assessed by a weighting method. Fig. 5 shows the weight loss and photographs of the two hydrogels after 12 h in oven at 50 °C (Fig. 5a) and in an open environment for 7 days (Fig. 5b). As shown in Fig. 5a, the hydrogel is soft and flexible before being placed in the oven. After been treated in the oven for 12 hours, the hydrogel became hard and the elasticity got worse, and the weight loss of the hydrogel without DMSO was more obvious. It can be found that the weight of the hydrogel without DMSO has changed by 81.4%, while the weight of the hydrogel prepared with DMSO has changed by 64.4%. It can be seen that the hydrogel with DMSO is relatively mild and the water retention performance is better. As shown in Fig. 5b, after storage in an open environment for 7 days, it can be found that about 16% of the initial weight was maintained for the hydrogel without DMSO, while the hydrogel prepared with DMSO could maintain about 80% of its initial weight. This is because DMSO, as an organic solvent, acts as a protective agent in the ionic conductive hydrogel and interacts with the water molecules in the hydrogel, thereby reduced the speed of water evaporation.

The conductivity of the hydrogel in an open environment was also investigated. As shown in Fig. 5c, the hydrogel with DMSO maintains a good conductivity after 7 days of storage in an open environment, while the hydrogel without DMSO was severely shrunken with almost no conductivity.

### Anti-freezing property test

**Fig. 6:**
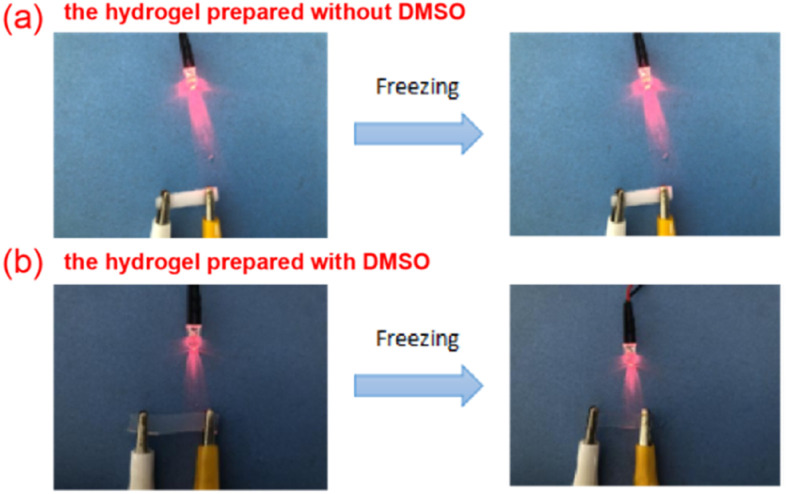
(a) PVA/CNC hydrogel without DMSO (b) PVA/CNC hydrogel with DMSO.

As shown in Fig. 6, the sample without DMSO was frozen and hardened completely, while the hydrogel with DMSO still maintained good mechanical properties. In addition, it can be seen from the brightness of the LED lamp that the antifreeze performance of hydrogel has a certain influence on its electrical conductivity. The conductivity of the hydrogel samples without DMSO decreased, while the conductivity of the hydrogel with DMSO was not significantly affected, because the anti-freezing performance of the hydrogel was improved after the addition of DMSO. The strong hydrogen bond between DMSO and water molecules could reduce the freezing point of water. Therefore, DMSO can effectively reduce the freezing point and inhibit the formation of ice crystals in the hydrogel.

### Sensing performance

**Fig. 7:**
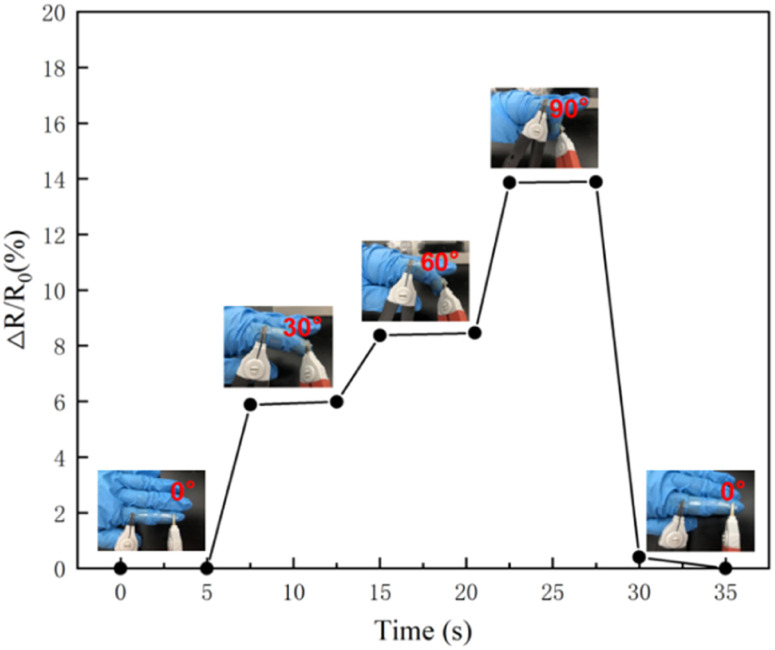
Changes in relative resistance when hydrogel adhered to human index finger at different angles.

As shown in Fig. 7, PVA/CNC ionic conductive hydrogel was bound to the index finger of the human body, and the index finger was bent at 0°,30°,60°,90°, and then slowly returned from 90° to 60°,30°, 0°, to test the relative resistance changes of hydrogel. Experiments show that the resistance changes when the index finger is bent at different angles. When the angle comes back to the original value, the resistance is the same. This indicates that the hydrogel has electrical stability and the resistance under different bending angles which can be monitored in real-time, reflecting that the prepared hydrogel has good strain sensitivity and electrical stability. It has the potential of preparing wearable electronic devices.

**Fig. 8:**
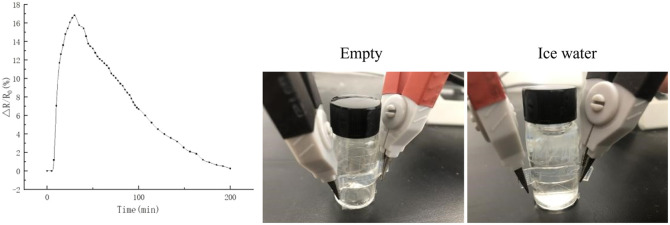
Hydrogels are composed of temperature sensors to test the temperature change of bottles containing ice water.

The hydrogel can be assembled into a simple temperature sensor, the hydrogel was immobilized on the outer surface of a glass vial, and a LCR digital bridge was connected at both ends to measure its resistance as a function of temperature (Fig. 8). At room temperature, the Δ*R*/*R*_0_ of the empty glass bottle is set to 0%. When ice water is added to the bottle, Δ*R*/*R*_0_ increases rapidly in a short time. As time goes on, the temperature of the glass bottle gradually returns to room temperature, and Δ*R*/*R*_0_ decreases gradually. According to the Fig. 8, when the temperature drops, the relative resistance of the hydrogel increases. When the temperature of the hydrogel drops to the lowest, the change value of the relative resistance of the hydrogel reaches its peak.

**Fig. 9:**
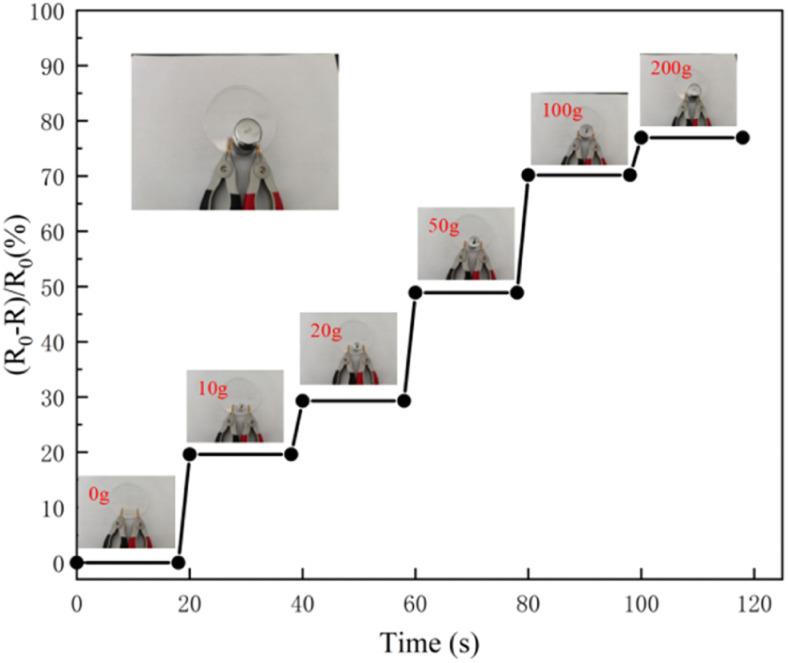
Relative resistance curves of PVA/CNC hydrogel under different pressures.

In order to study the sensitivity of the hydrogel under different weight compressions, the change of relative resistance with pressure was measured. The hydrogel sensor exhibited quantifiable responsiveness upon compression from 10 g to 100 g. It can be seen from Fig. 9 that the heavier the hydrogel weight, the greater the relative resistance change. This shows that the detection of slight force may widen the ranges of potential applications of the hydrogel.

### The touch performance of hydrogel

**Fig. 10:**
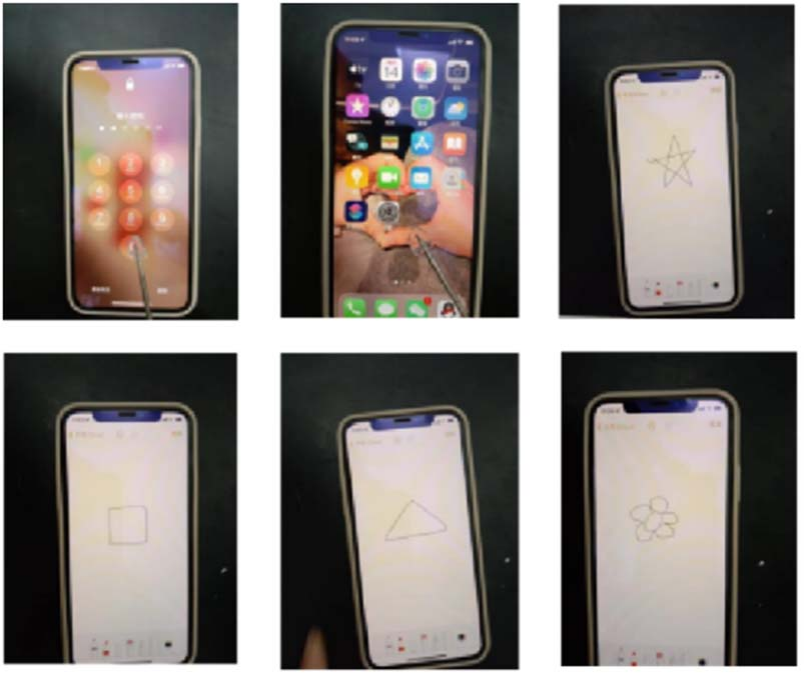
Hydrogel is assembled into a touch electronic pen, which can unlock the phone.

As shown in Fig. 10, PVA/CNC hydrogel can be assembled to form an electronic touch pen, which can be used to unlock the phone and do some drawings on the screen. This is because the hydrogel creates a coupling capacitance when it comes into contact with the screen surface. The position of the touch point is precisely calculated by the controller in the phone. This reflects the potential of ionically conductive hydrogels in human–computer interaction.

### Thermal stability analysis

**Fig. 11:**
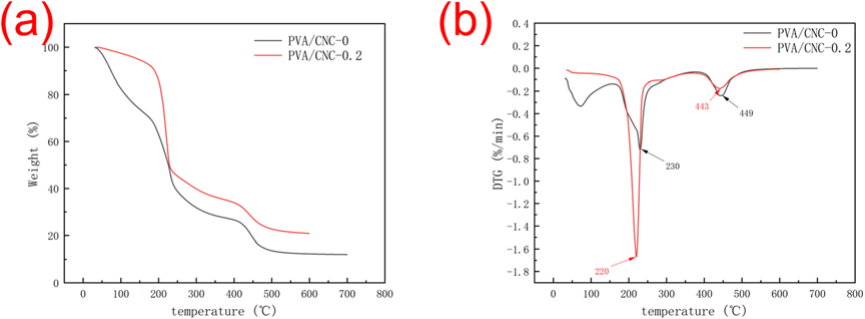
(a)TGA and (b) DTG curves of pure PVA hydrogel and PVA hydrogel reinforced by 0.2 wt% CNC.

The onset temperature (*T*_onset_) and the temperature at maximum decomposition rate (*T*_max_) are shown in Table 2. The first stage degradation of the PVA/CNC hydrogel is between 175–300 °C. The first degradation region is due to the pyrolysis of CNCs and the degradation of PVA. When CNC is added to it, it can be seen that the sample mass loss tends to decrease. The first stage of degradation mainly involves dehydration reactions and the formation of volatile products. The mass loss in the second stage occurs above 380 °C and involves the decomposition of carbonaceous materials. As displayed Fig. 11a, the amount of carbon residue in the PVA/CNC hydrogel will increase compared with pure PVA. It can be seen that the hydrogel with 0.2 wt% CNC had onset thermal degradation temperature of approximately 196 °C, compared with 188 °C for pure PVA hydrogels, respectively. This may be due to the strong interaction between the hydroxyl groups of PVA macromolecules and the hydroxyl groups on the surface of CNC particles.

**Table 2:** Onset temperature (*T*_onset_) and temperature at maximum decomposition rate (*T*_max_) of the PVA/CNC composites

Sample	*T*_onset_ (°C)	*T*_max_(°C)
PVA/CNC-0	188	230
PVA/CNC-0.2	196	220

## Conclusions

In summary, a one-pot method was used to prepare PVA/CNC hydrogels. The prepared ion-conducting hydrogel exhibits a three-dimensional network porous structure. With the increase of CNC concentration, the density of the hydrogel increases, and the mechanical properties of the hydrogels are enhanced. The PVA/CNC-0.2 hydrogel exhibits a high tensile strength of about 750 kPa and an elongation at break of 410.47%. While the content of CNC continues to increase, it shows a downward trend. Compared with the hydrogel without DMSO, the weight loss of the hydrogel prepared with DMSO was reduced by 17%. It can be seen that the water retention performance of the hydrogel will be enhanced after the addition of DMSO. The prepared ionically conductive hydrogel can also be assembled into a self-powered device with a voltage of up to 0.808 V. The hydrogel can also be used in the field of human–computer interaction, which can be used to unlock mobile phones or tablets and do some drawings.

## Author contributions

Huang Xinmin: conceptualization, resources, supervision, funding acquisition, writing – review & editing. Ao Xiang: methodology, investigation, formal analysis, data curation, writing – original draft. Yang Lianhe: validation. Ye Jing: investigation, visualization. Wang Chengwei: project administration, formal analysis.

## Conflicts of interest

There are no conflicts of interest to declare.

## Supplementary Material
